# Supplementary material for: Vimentin regulates mitochondrial ROS production and inflammatory responses of neutrophils
Source: Front Immunol. 2024 Jul 30;15:1416275. doi: 10.3389/fimmu.2024.1416275 (PMC11319119; doi:10.3389/fimmu.2024.1416275)
Supplement: Supplementary file 1 [file DataSheet_1.pdf]

Supplementary Figures

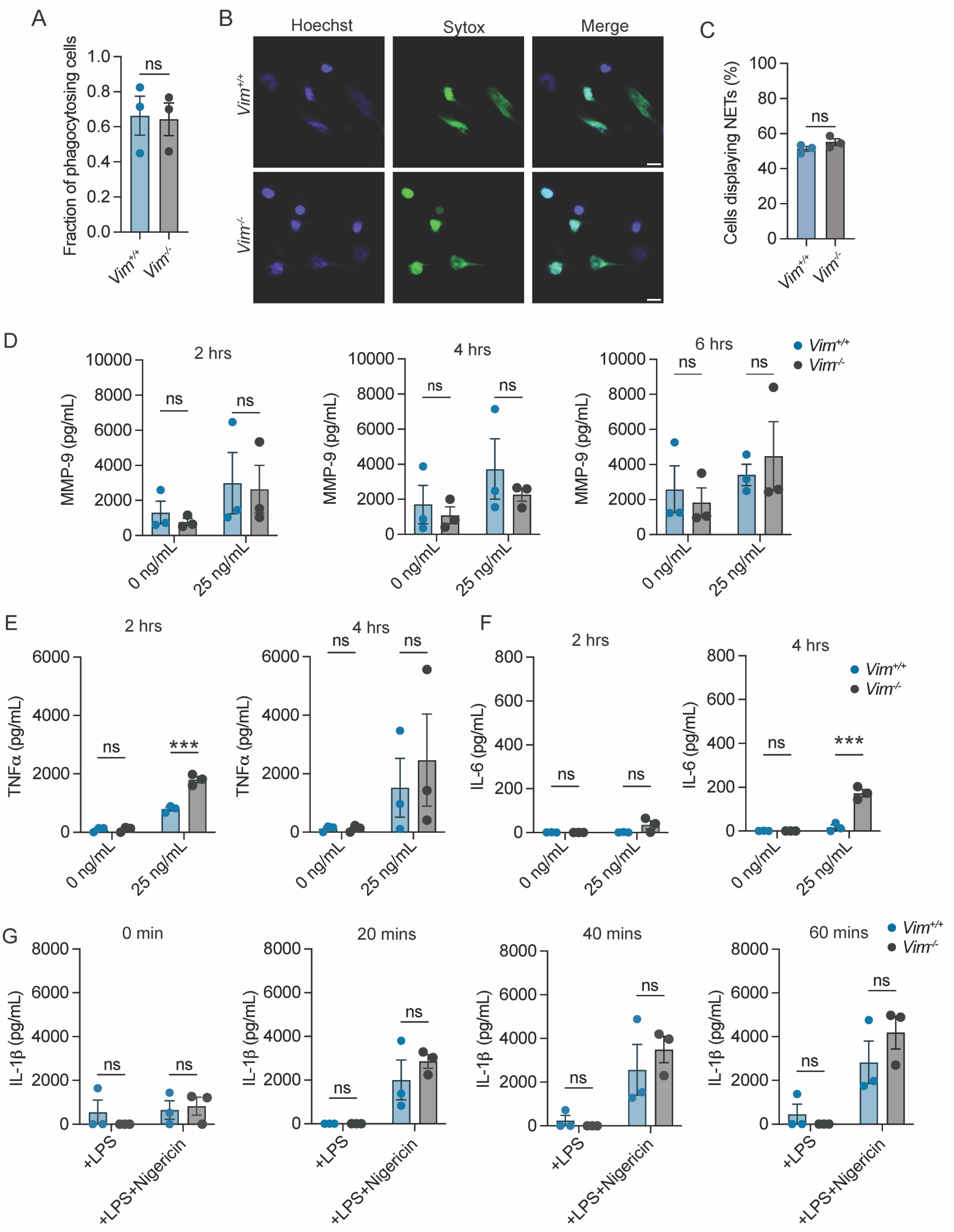

Figure S1. Neutrophil functions that are not affected by vimentin.

A) Fraction of phagocytosing cells. B) Representative images of NET formation induced by A23187. Green, extracellular DNA; blue, all DNA. Scale bar: 10 μm. C) Quantification of percentage of cells displaying NETs in *Vim*$^{+/+}$ and *Vim*$^{-/-}$ neutrophils from images represented in B. D) Levels of MMP-9 (pg/mL) released from neutrophils treated with LPS (0 and 25 ng/mL) at 2, 4, and 6 hours by ELISA. E-F) Quantification of TNFα (E) or IL-6 (F) produced by *Vim*$^{+/+}$ and *Vim*$^{-/-}$ neutrophils in response to LPS stimulation at 2 and 4 hours. G) Quantification of IL-1β produced by *Vim*$^{+/+}$ and *Vim*$^{-/-}$ neutrophils in response to treatment with LPS alone or LPS and nigericin at 0, 20, 40, and 60 minutes. Data are mean ± SEM from three independent experiments with 5 *Vim*$^{+/+}$ or 6 *Vim*$^{-/-}$ cell lines. Dots are independent experiments. ns, not significant by paired t-test (A, C) or two-way ANOVA with Sidak's *post hoc* test (D-G).

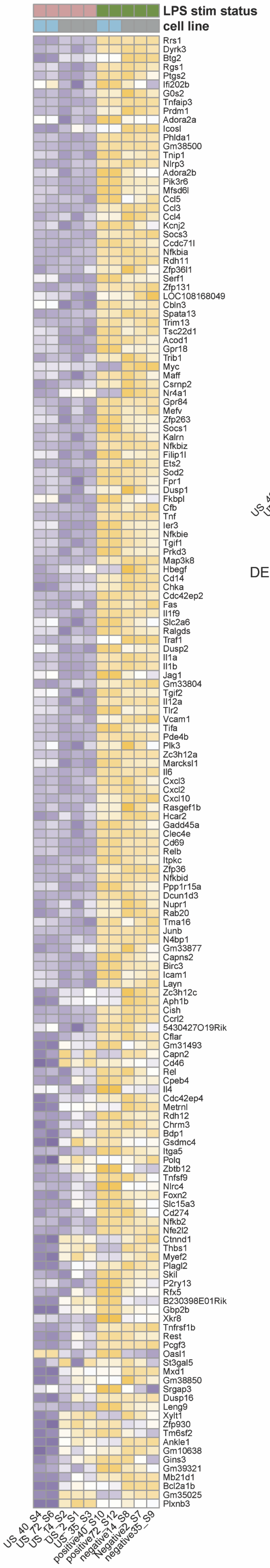

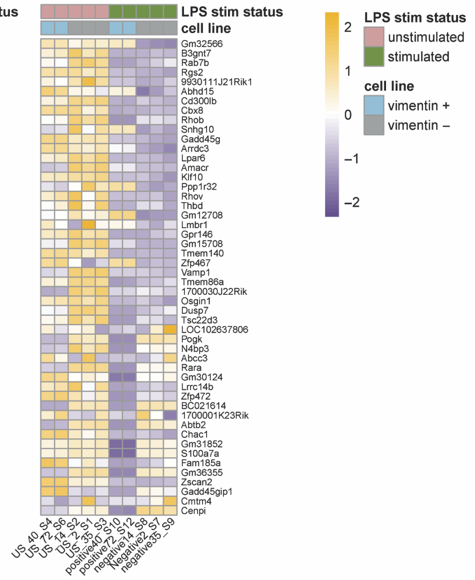

DE genes: log2(FC) > 1 or log2(FC) < -1 and p-adj < 0.05
(scaled expression)

Figure S2. Heatmap of gene differential expression.

Heatmap showing differential expression genes with $\log_2$(fold_change) > 1 or $\log_2$(fold_change) < -1 with adjusted p-value < 0.05 in all conditions.

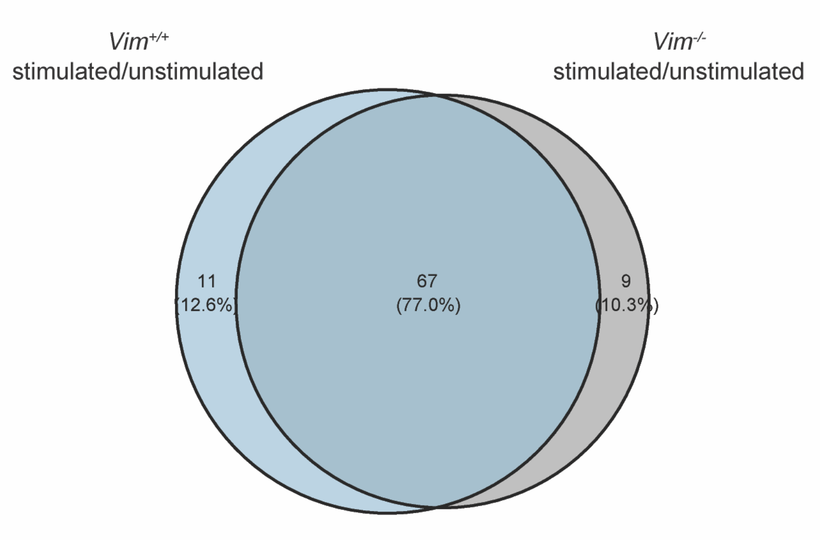

Figure S3. LPS stimulation affects similar genes between *Vim*$^{+/+}$ and *Vim*$^{-/-}$ for TNFα signaling via NF-κB hallmark.

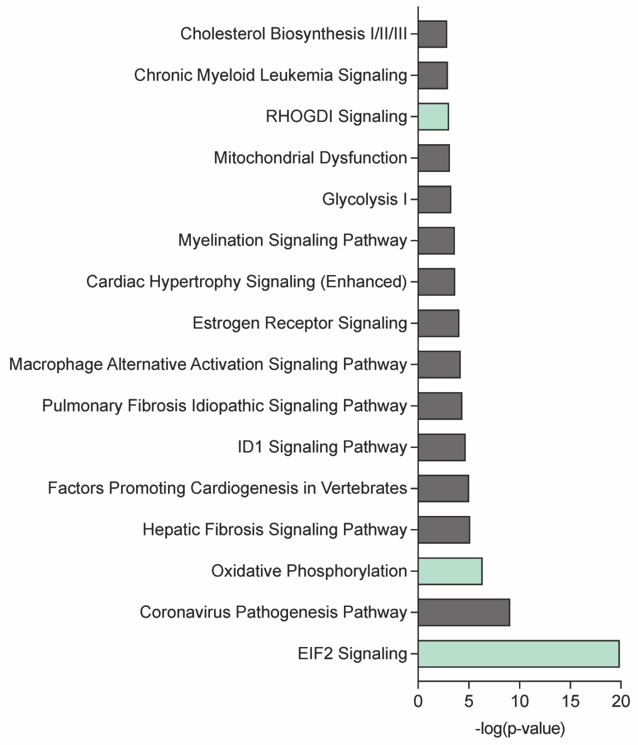

Figure S4. Bar graph showing pathways altered in *Vim*$^{-/-}$ compared to *Vim*$^{+/+}$ neutrophils. The top 20 pathways affected with the highest -log(p-value) from Ingenuity Pathway Analysis were selected and the pathways with the z-score > 2 or z-score < -2 were considered significant. Gray, upregulated in *Vim*$^{-/-}$ cells; green, downregulated in *Vim*$^{-/-}$ cells.

Supplemental Material 1. Raw RNA-Seq data.

Supplemental Material 2. Pathways analysis data from Ingenuity Pathway Analysis.
